# Differences in Activation Area Within Brodmann Area 2 Caused by Pressure Stimuli on Fingers and Joints: In Case of Male Subjects

**DOI:** 10.1097/MD.0000000000001657

**Published:** 2015-09-25

**Authors:** Mi-Hyun Choi, Hyung-Sik Kim, Ji-Hye Baek, Jung-Chul Lee, Sung-Jun Park, Ul-Ho Jeong, Seon-Young Gim, Sung-Phil Kim, Dae-Woon Lim, Soon-Cheol Chung

**Affiliations:** From the Department of Biomedical Engineering (M-HC, H-SK, J-HB, J-CL, S-JP, U-HJ, S-YG, S-CC), Research Institute of Biomedical Engineering, College of Biomedical & Health Science, Konkuk University, Chungju; Department of Human and Systems Engineering (S-PK), Ulsan National Institute of Science and Technology, Ulsan; and Department of Information and Communication Engineering (D-WL), Dongguk University, Seoul, South Korea.

## Abstract

In this study, a constant pressure stimulus was applied on the 3 joints (first [p1], second [p2], and third [p3] joints) of 4 fingers (index, middle, ring, and little fingers), and the activation areas within Brodmann area 2 (BA 2) were compared for these different fingers and joints by using functional magnetic resonance imaging.

Eight healthy male college students (25.4 ± 1.32 years) participated in the study. Each session was composed of 3 blocks, and each block was composed of a Control phase (30 seconds) and a Pressure phase (30 seconds). No pressure stimulus was applied in the Control phase, during which the subjects would simply lay comfortably with their eyes closed. In the Pressure phase, a pressure stimulus was applied onto one of the joints of the selected finger.

For each finger and joint, BA 2 areas activated by the pressure stimulus were extracted by the region of interest method. There was a significant difference in the activation areas for the different fingers (*P* = .042) as well as for the different joints (*P* = .050). The activation area decreased in the order of the little, index, and middle fingers, as well as in the order of p1, p3, and p2.

## INTRODUCTION

Many studies have examined, using functional magnetic resonance imaging (fMRI), the neurological changes in the primary somatosensory area (S1) caused by the application of various tactile stimuli.^1–7^ Such studies provide important insights into not only the normal organization of sensory cortices, but also into how neural systems are affected by disease or damage and how they may change over time during remodeling and with intervention.^8–10^ Data relating to the application of various tactile stimuli may also be useful in longitudinal studies designed to monitor cerebral plasticity and reorganization, for example in sensorimotor recovery after neurosurgery, cerebral ischemia,^11^ or in understanding mechanisms of supraspinal pain processing.^12^

The S1 area can be divided into Brodmann areas (BA) 1, 2, and 3. BA 3 is activated by vibrations, pressure, and most other tactile stimuli; BA 2 is activated by pressure, joint position, and complex touch; and BA 1 usually responds to vibrotactile stimuli.^13^

The above-mentioned studies examined changes in activation areas within the somatosensory area, especially in BA 3, in response to vibration stimuli on fingers.^1–7,14^ Differences in the activation areas within BA 3 were observed across joints^7,14^ and across fingers when a vibration stimulus was applied to the first joint.^1–6^ These studies point to the consistent interest in BA 3 activation areas in relation to application of vibration stimuli to fingers and joints.

Other studies have focused on how pressure stimuli lead to changes in the activated area of the somatosensory area. For example, some studies observed changes in BA 3 activation areas upon application of a pressure stimulus to the first joints of the fingers.^15–17^ Specifically, van Westen et al^17^ found that when the pressure stimulus was applied to the first joint of the little finger, the activation area was larger than when the pressure stimulus was applied on the middle finger. Overduin and Servos^15,16^ found that the activation area triggered by the index finger was larger than that triggered by the little finger when the same pressure stimulus was applied to the first joints of both these fingers. According to previous studies, activation areas within BA 3 decrease in the order of the index, little, and middle fingers when a pressure stimulus is applied to the first joints of each of these fingers. Although some differences have been reported in BA 3 activation areas triggered by pressure stimuli applied to different fingers, no study seems to have addressed differences in activation areas for different joints. Moreover, previous researchers focused only on BA 3, referred to as the “primary somatosensory cortex,” and not on BA 2, which is known to respond well to pressure stimuli. As such, further research is needed in order to investigate activation areas in the case of pressure stimuli applied to fingers. In other words, it is important to conduct research on differences in activation areas for pressure stimuli applied to different joints as well as on those for pressure stimuli applied to different fingers and to expand the focus from BA 3 to BA 2, which has high sensitivity to pressure stimuli.

The present study was conducted focusing on the following 2 points in order to overcome the limitations of preceding studies. First, to date, preceding studies have compared activation areas in response to vibration and pressure stimuli applied to the first joints of different fingers, whereas in the present study, we have carried out the comparison between fingers as well as between joints. Second, most preceding studies focused on BA 3 of the somatosensory area when vibration and pressure stimuli were applied to fingers, whereas the present study has focused on BA 2, which has been reported as the most sensitive to pressure stimuli.

Accordingly, in this study, pressure stimuli were applied to the 3 joints (first [p1], second [p2], and third [p3] joints) of 4 fingers (index, middle, ring, and little finger) and the resulting activation areas within BA 2 were compared between these different fingers and different joints.

## METHODS

To date, there have been no reports on the differences in activation patterns in response to tactile stimuli applied to different joints on the fingers. As a first step toward accurately assessing these differences, a preliminary experiment was first performed to assess the contribution of sex with a higher sensitivity because results of a preceding study showed differences in sensitivity to tactile stimuli by sex.^18^ Specifically, a preliminary experiment including 5 men (23.2 ± 1.2 years) and 5 women (22.6 ± 0.7 years) was performed to determine their ability to differentiate between pressure stimuli by joint. A pressure stimulus (8.5 psi) was applied to the 3 joints of the index finger with the intention to study the contribution of sex with a higher sensitivity. We found that men more accurately differentiated pressure stimuli by joint than women did. Therefore, 8 healthy male college students (25.4 ± 1.32 years) participated in the main study as subjects. None of them reported having a history of psychiatric or neurological disorders. The overall procedure was explained to all of them, and they provided informed consent for the procedure. All experimental procedures were approved by and performed under the regulations of the Institutional Review Committee of Korea University (KU-IRB-11-46-A-1).

**FIGURE 1:**
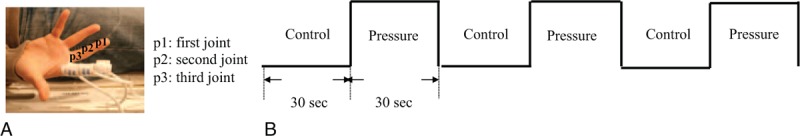
(A) Pressure stimulator and (B) experimental design.

Pressure stimuli were applied using an MR-compatible pressure stimulator.^19^ This method stimulates finger joints by injecting air into a noninvasive blood pressure cuff (M1866A, Philips, The Netherlands) (Figure 1A). The air pressure generated from the air pump is delivered to the cuff, which measures 6.4 cm × 2.5 cm, via a 7 m air tube. E-Prime software (Psychology Software Tools, Inc. Sharpsburg, PA) was used to control parameters such as the pressure intensity and time. The functional images were obtained while applying a constant pressure (8.5psi) on a joint (p1, p2, and p3) of each of the 4 right fingers (index, middle, ring, and little fingers).

Three pressure stimulators were attached to the 3 joints (p1, p2, and p3) of an arbitrarily selected finger (index, middle, ring, or little finger) (Figure 1A). Figure 1B shows the paradigm for the fMRI experiments. A session consisted of 3 blocks, and each block was composed of a Control phase (30 seconds) and a Pressure phase (30 seconds). No stimulus was applied in the Control phase, during which the subjects would simply lie comfortably with their eyes closed for 30 seconds. In the Pressure phase, a 30-second pressure stimulus was applied onto one of the joints (p1, p2, or p3) of the selected finger (index, middle, ring, or little finger). Because there were 3 Pressure phases in the 1 session, all 3 joints were subjected to pressure stimuli. Each session was repeated thrice for each finger. During these 3 sessions, the 3 joints of the selected finger were subject to pressure stimuli for a total of 3 times. The remaining 3 fingers were subjected to the same procedures. The order of providing the stimuli for each finger and joint was counterbalanced, and a 5 min break was given between sessions. All subjects participated in 12 sessions (3 sessions/finger **×** 4 fingers = 12 sessions). They wore headsets and kept their eyes closed to eliminate audiovisual influences. The subjects were asked to minimize hand and head movements.

Images were scanned with a 3-T MRI system (Magnetom TrioTim, Siemens Medical Systems, Erlangen, Germany) using a standard 32-channel head coil. Anatomical images were obtained using a T1-weighted 3D-MPRAGE sequence with a repetition time (TR) of 1900 ms, echo time (TE) of 2.48 ms, flip angle of 9°, field of view (FOV) of 200 mm, and spatial resolution of 0.8 **×** 0.8 **×** 1 mm^3^. Functional images were obtained using a T2∗-weighted gradient echo-planar imaging sequence with TR of 3000 ms, TE of 30 ms, flip angle of 90°, FOV of 192 mm, slice thickness of 2 mm, and in-plane resolution of 1.5 **×** 1.5 mm^2^.

The fMRI data were analyzed using SPM 8 (Wellcome Department of Cognitive Neurology, London, UK). All functional images were aligned with the anatomical images from the study using affine transformation routines built into SPM 8. The realigned scans were coregistered to a subject's anatomical images obtained within each session and normalized to SPM8's template image that uses the space defined by the Montreal Neurological Institute. Motion correction was performed using sinc interpolation. Time-series data were filtered with a 240-second high-pass filter to remove artifacts because of cardiorespiratory and other cyclical influences. The functional map was smoothed with a 3 mm isotropic Gaussian kernel before statistical analysis. Statistical analysis was performed both individually and as a group using the general linear model and the theory of Gaussian random fields incorporated into SPM 8. Statistical parametric maps with the *t*-statistic were computed. The analysis of data for individual subjects was performed at a significance threshold of *P* < 0.05 corrected for multiple comparisons. Furthermore, random effect analysis was used to compute group activation.

The region of interest method was used to observe activation areas only within BA 2 upon application of pressure stimuli. The subtraction method (Pressure phase–Control phase) was used to extract from BA 2 the number of activated voxels for stimuli on each finger and joint by subject, and activation areas were then calculated (activation area [mm^2^] = number of activated voxels **×** 1.5 mm **×** 1.5 mm). Statistical differences in activation areas for the different fingers and different joints were determined by performing repeated-measures two-way ANOVA (PASW Statistics 18). Moreover, in order to examine if there was a statistically significant difference between activated BA 2 areas activated by stimuli to each joint (p1, p2, or p3) by finger and activated BA 2 areas in response to stimuli by joint in each finger (index, middle, and little fingers), repeated-measures one way ANOVA (PASW Statistics 18) was performed for each area. In addition, a pairwise analysis revealed significant differences in activation areas for the different fingers and different joints.

## RESULTS

**FIGURE 2:**
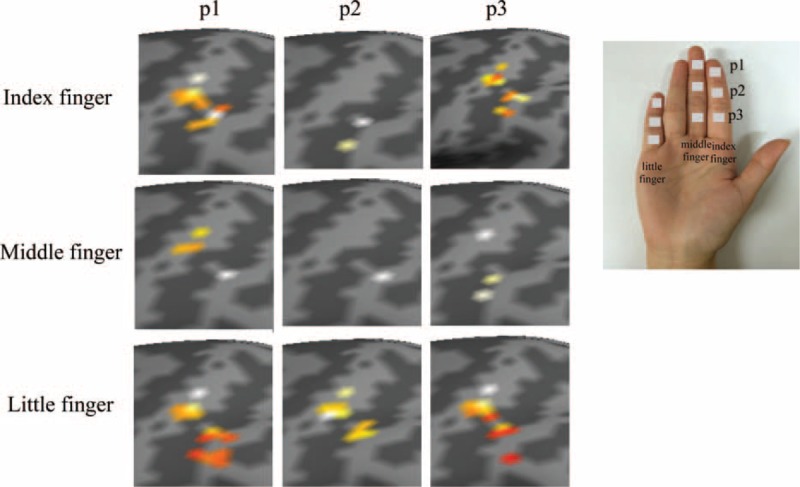
Imaging data of the activation of BA 2 by finger and joint in response to pressure stimuli (p1: first joint, p2: second joint, p3: third joint). BA 2 = Brodmann area 2.

Table 1 shows BA 2 activation areas (mean ± SE mm^2^) following application of pressure stimuli to the different fingers and joints for each subject. In addition, the group analysis results of all subjects in response to pressure stimuli are shown in Figure 2 according to finger and joint. In the case of stimuli applied to the joints of the ring finger, activation areas in BA 2 were observed only for 3 out of 8 subjects. This result was regarded as insignificant and was thus excluded from further analysis. Repeated-measures two-way ANOVA revealed significant differences in activation areas between different fingers (*P* = 0.042) and between different joints (*P* = 0.050) (Table 2).

**TABLE 1:**
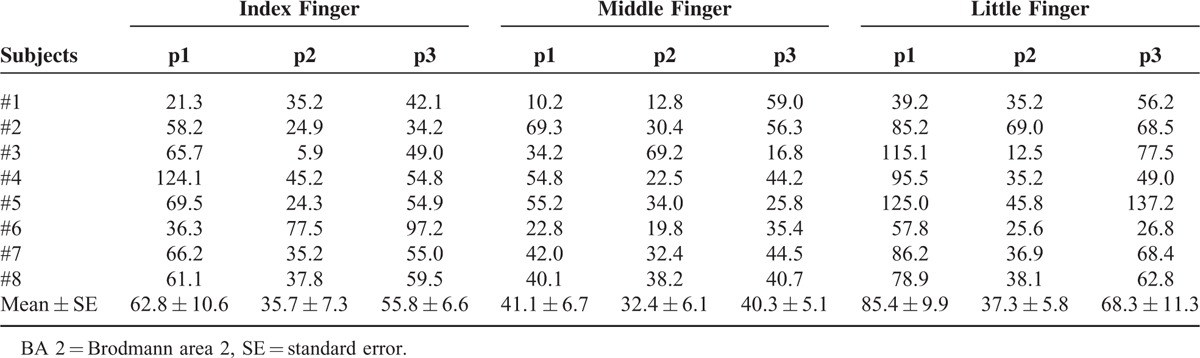
Activation Areas (mm^2^) Within BA 2 for Pressure Stimuli Applied to Fingers (Index, Middle, and Little Fingers) and Joints (First [p1], Second [p2], and Third [p3] Joints) for Each Subject

**TABLE 2:**
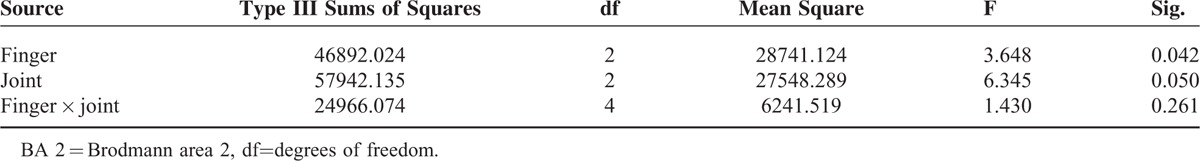
Repeated-Measures Two-Way ANOVA for Activation Areas Within BA 2 With Fingers and Joints as Independent Variables

**FIGURE 3:**
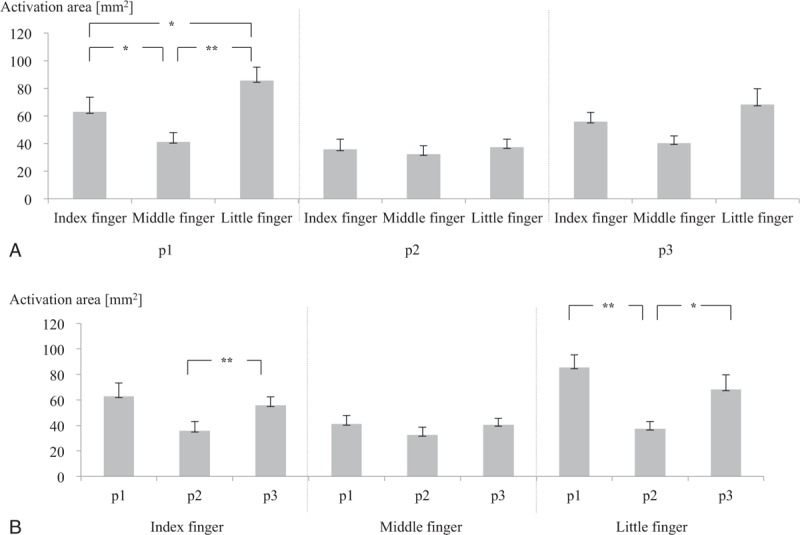
Activation area within BA 2 for (A) fingers and (B) joints (p1: first joint, p2: second joint, p3: third joint) (∗*P* < 0.05; ∗∗*P* < 0.01). BA 2 = Brodmann area 2.

With regard to the stimulated finger, the activation area in BA 2 was largest following stimulation of the little finger, followed by the index finger and finally the middle finger. Statistical analyses of activated BA2 areas by finger identified a significant difference by finger in case of p1 (*P* < 0.001), whereas no significant differences by finger were identified in case of p2 and p3. Pairwise analysis of p1 found significant differences between the index finger and the middle finger (*P* = 0.032), between the index finger and the little finger (*P* = 0.040), and between the middle finger and the little finger (*P* = 0.001) (Figure 3A).

With regard to the stimulated joint, the activation area in BA 2 was largest in response to stimulation of p1, followed by p3 and finally p2. Statistical analysis of activated BA 2 areas by joint showed a significant difference by joint in the index finger (*P* = 0.049) and the little finger (*P* = 0.001), whereas the middle finger exhibited no significant difference by joint. Pairwise analysis identified significant differences between p2 and p3 (*P* = 0.002) in the index finger, and between p1 and p2 (*P* = 0.004), and between p2 and p3 (*P* = 0.028) in the little finger (Figure 3B).

## DISCUSSION

A constant pressure stimulus was applied on the 3 joints (p1, p2, and p3) of 4 fingers (index, middle, ring, and little finger), and the activation areas within BA 2 for the different fingers and different joints were compared using fMRI.

In our study, it was found that the activation area within BA 2 was largest when the stimulus was applied to the little finger, followed by the index finger and finally the middle finger. Our results are consistent with those of a study by Peters et al,^20^ who determined the tactile sensitivity of the first joints of the 5 fingers through a subjective assessment and reported higher sensitivity for smaller fingers. This is because the smaller the finger, the more elaborate the distribution of the sensory receptors, which in turn leads to higher tactile sensitivity. The little finger, being the smallest of the 5 fingers, has the highest sensitivity. However, our result is inconsistent with the results of some previous work,^15–17^ in which the activation area (BA 3) decreased in the order of the index, little, and middle fingers. The discrepancies between the results of our study and those of previous studies^15–17^ on the activation area according to finger stimuli can be explained by one of the following reasons. In the previous studies, it was difficult to observe high levels of sensitivity to specific stimuli (pressure), as the focus was on BA 3, which is an area that responds to tactile stimuli such as vibrations and pressure. However, the present study observed the activation area within BA 2, which is an area that usually responds to pressure stimuli, and this is likely to have produced results with higher sensitivity. The differences could also have arisen from the choice of experimental method (the type of stimuli, strength, time, etc.). Nevertheless, further studies are required to determine the exact causes.

Because past research involved providing stimuli only to the first joint of each finger, it was impossible to compare the differences in activation areas between different joints. In contrast, the present study found a decrease in the activation area within BA 2 in the order of p1, p3, and p2. Weinstein^21^ applied pressure stimuli of various strengths to the joints of 5 fingers and measured the tactile sensitivity of subjects based on a subjective assessment. The p1 was found to have the highest sensitivity, followed by the p3 and finally the p2; this result is in agreement with the results of our study. This implies that the activation area within BA 2 accurately reflects the tactile sensitivity to pressure stimuli on joints.

In a study of tactile sensitivity, Weinstein^21^ found that the ring finger was less sensitive than the other 4 fingers. This may explain why we observed some inconsistencies when the pressure stimulus was applied to the ring finger in the present study.

In conclusion, the largest activation area within BA 2 was associated with the little finger in the case of pressure stimuli applied to different fingers, and with the p1 in the case of pressure stimuli applied to different joints. The results of previous work conducted with a focus on tactile sensitivity^20,21^ were verified from a neurological perspective. This study showed that differences in tactile sensitivity to pressure stimuli on different fingers and joints can be reflected in changes in the activation area within BA 2. These findings are expected to serve as a basis for neurological research on the effects of pressure stimuli provided to fingers and joints on the S1 (BA 2).

The results of the present study could be referred to while carrying out a comparison between normal subjects and abnormal subjects for studies of somatosensory activation in clinical trials (eg, studies on cerebral plasticity and reorganization following neurosurgery or ischemic brain injury). In general, pressure stimuli have been used mainly to diagnose patients who have neurological disorders that affect tactile sensation. However, tactile stimuli have been applied only to the first joint of the index finger for the diagnosis of patients. According to the results of the present study, the p1 of the little finger, which showed the largest activation in response to pressure stimuli among the 3 joints of the 4 fingers, would be useful in the diagnosis of neurological disorders in the somatosensory area. However, it is necessary to conduct various further studies to verify this potential clinical application.

In the present study, we applied pressure stimuli to each of the 3 joints of the 4 fingers, compared the activation areas in BA 2 in the S1 area by finger and joint, respectively, and then presented the results. Despite not being presented in the results, data on the activation areas in BA 1 and BA 3 were also extracted by finger and by joint. However, activation areas appeared in only 2 out of 8 subjects in total. In other words, the BA 2 area that dominantly receives information of pressure tactile stimuli clearly showed activation by finger and by joint, whereas BA 1 and BA 3 did not show significant activation. It is possible that these results may have been influenced by the small number of subjects in the present study. It is necessary to increase the sample size to analyze activation patterns not only in the BA 2 area, but also in BA 1 and BA 3, and we also need to increase the sample size for a comparison with BA 2. In addition, it is necessary to study differences by sex and by age, and to perform a comparative study between normal groups and patient groups, which would create a more solid basis for clinical application.
